# *Dejian* Mind-Body Intervention on Depressive Mood of Community-Dwelling Adults: A Randomized Controlled Trial

**DOI:** 10.1093/ecam/nep043

**Published:** 2011-06-23

**Authors:** Agnes S. Chan, Mei-chun Cheung, Wilson J. Tsui, Sophia L. Sze, Dejian Shi

**Affiliations:** ^1^Department of Psychology, The Chinese University of Hong Kong, Shatin, Hong Kong; ^2^Integrative Neuropsychological Rehabilitation Centre, The Chinese University of Hong Kong, Hong Kong; ^3^Henan Songshan Research Institute for Chanwuyi, Henan 452470, China; ^4^Institute of Textiles and Clothing, The Hong Kong Polytechnic University, Hong Kong

## Abstract

The present study evaluated the effectiveness of a short-term mind-body intervention program on improving the depressive mood of an adult community sample. Forty adult volunteers with various degrees of depressive mood were randomly assigned to the experimental group (*Dejian* Mind-Body Intervention, DMBI) and control group (Cognitive-Behavioral Therapy, CBT). For each group, a total of four 90-min weekly sessions were conducted. Treatment-related changes were measured using the Beck Depression Inventory (BDI-II), an electroencephalographic indicator of positive affect (i.e., prefrontal activation asymmetry), and self-report ratings on physical health. Results indicated that both the DMBI and the CBT group demonstrated significant reduction in depressive mood. However, among individuals with moderate to severe depressive mood at baseline, only those in the DMBI but not the CBT group showed significant reduction in depressive mood. Besides, only the DMBI group demonstrated a significant increase in prefrontal activation asymmetry, suggesting increase in positive affect. While most psychological therapies for depressive mood normally take several months to show treatment effect, the present findings provided initial data suggesting that the DMBI was effective in improving depressive mood of community adults after 1 month of training.

## 1. Introduction

Mind-body intervention emphasizes the interaction among the brain, the mind and the body. The fundamental assumption is that individuals have internal ability to change their own thoughts and behaviors, and to enhance their emotional and physical health. The concept that the mind plays an important role in the improvement of health is a core component of traditional Chinese medicine that dates back at least 2000 years in China. With the emphasis that treatment should be holistic in nature including moral, spiritual and environmental factors, Chinese have developed different kinds of mind-body intervention including *qigong*, *tai chi*, relaxation training, thought training (e.g., *Chan* or Zen) and dietary modification.

Although mind-body interventions have been practiced for centuries in the East, Western countries have studied this approach more extensively than the East in the past 20 years. There have been scientific and clinical studies which demonstrated the therapeutic effects of mind-body intervention in clinical and normal populations [1]. In clinical practice, an increasing number of empirical studies have reported positive effects of mind-body training on different mental and physical problems, such as depression [2–4], anxiety [5], obsessive-compulsive disorder [2, 6], insomnia [7], chronic pain [8], tension headaches [9], hypertension [10, 11], cardiac diseases [12], type II diabetes mellitus [13], irritable bowel syndrome [14], cancer [15] and viral infection [16]. In a recent study conducted by Shapiro and colleagues [4], a 20-session Yoga (a form of mind-body intervention) program was found to have effectively elevated the mood and improved psychological well-being of patients with depression in partial remission. Apart from encouraging effects on mental [17, 18] and physical health [19–22] in clinical samples, some empirical data have also suggested that mind-body training has a positive impact on cognitive, emotional and psychological problems in normal population [23–30]. For instance, women who did mind-body training (i.e., *Tai Chi*) for 4 months showed significantly reduced anger and total mood disturbance, as compared with those who did aerobic exercises [31]. Another study showed that after 3 months of mind-body training, older adults had significant reduction in somatic and psychological depressive symptoms [24]. Similar effects were also observed on primary school children who demonstrated improved academic performance and reduced behavioral problems after 4 months of mind-body training [23].

**Figure 1:**
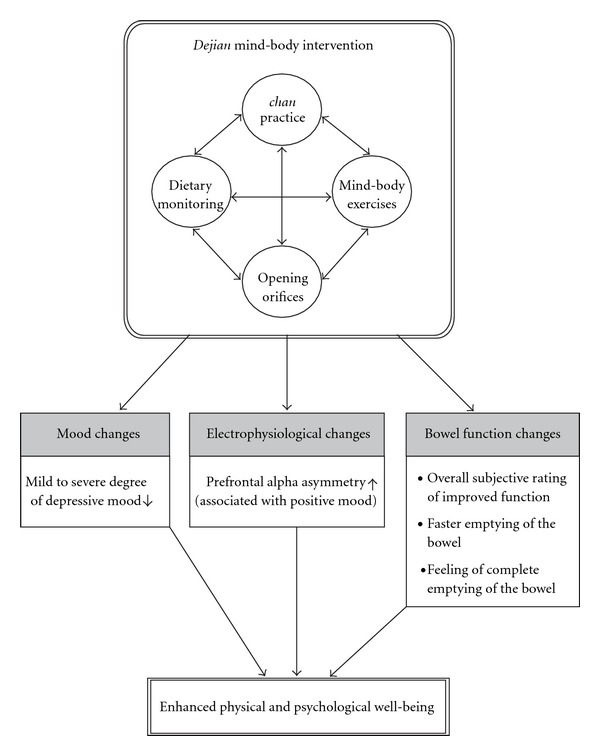
Illustration of the positive effects of DMBI on mood, neuro-electrophysiological state and bowel function.

While most of the empirical studies on mind-body intervention reported positive effects after several months of intervention, the purpose of the present study was to examine whether a four-session mind-body intervention administered over 1 month can have a positive effect on emotion. The *Dejian* Mind Body Intervention (DMBI) is a newly developed mind-body intervention by the first and the last authors. The characteristic of this mind-body intervention is that its development was based upon traditional *Shaolin Chan* (i.e., Zen) practice which consists of four components: (i) *Chan* practice, (ii) mind-body exercises, (iii) dietary monitoring and (iv) opening orifices; which can in turn improve physical and psychological health (Figure 1). Clinical observation demonstrated that this intervention brings about improvements in mood and physical health after 1 month of practice for patients with different problems such as depressive mood, back pain and behavioral problems. The treatment effects on some cases have been reported in the book *Dejian Mind-Body Intervention* [32]. For example, the DMBI was effective in improving the coronary heart disease of a patient that was resistant to Western medical treatment; in improving the motor functioning of a patient with motor neuron disease; in improving the functioning of a patient with spinal injury; in improving the condition of a patient with head injury and in improving the condition of less severe problems such as lower back problem and upper respiratory tract infection. Given these encouraging clinical observations, the aim of the present study was to study its effect empirically. Cognitive-behavioral therapy (CBT), which is considered one type of mind-body intervention by the National Center for Complementary and Alternative Medicine [33] and proven to be effective in reducing depressive mood [34–36], was used as a control condition. In addition to studying the effect of the DMBI on the mood of community adults with depressive mood, we also examined its effect on brain state using a quantitative electroencephalographic (QEEG) measure. The QEEG of prefrontal activation asymmetry at resting condition has been reported to be related to dispositional affect where relative left-sided prefrontal activation is related to positive affect [37]. Findings from a previous study demonstrated that individuals showed a significant leftward shift of resting prefrontal activation asymmetry after participating in a mind-body treatment [38]. Thus, the current study employed this measure as a more objective indicator of positive mood change related to the DMBI.

## 2. Methods

### 2.1. Participants

Participants were recruited from a public lecture on mind-body intervention. Individuals who volunteered to participate completed and returned a short questionnaire on their emotional and physical conditions. Individuals with any one of the following conditions were excluded from the study: (i) history of head injury, learning disability, seizure, stroke, other central nervous system disease or any endocrinopathy not stabilized by medication; (ii) history of psychosis or mania. A total of 40 adults voluntarily participated in this study, who met the inclusion criteria and demonstrated some symptoms of depressive mood. The group size was based on a review paper on previous studies using group CBT for depression in which the sample size was at least 10 with significant difference found [36]. The participants were aged from 25 to 64 years and have attained at least 9 years of formal education, and were randomly assigned into either the DMBI or CBT group using the block randomization method. As blocked by gender, an equal number of 15 females and 5 males were arranged into each treatment group. Given that the majority of the participants were aged between 40 and 50 years with high school level or above, the two groups after randomization were matched on age [*t*(19) = 0.30, *P* = .76] and education [*t* (19) = 0.18, *P* = .86]. Demographic characteristics of participants are presented in Table 1.

### 2.2. Assessment

The study was performed in accordance with the Helsinki Declaration of the World Medical Association Assembly. The experimental procedure was approved by the Joint CUHK-NTEC Clinical Research Ethics Review Committee. Informed consent was obtained from all participants. Each participant was administered the baseline assessment within 2 weeks before the intervention, and the post-assessment within 2 weeks after the intervention.

During the assessment, participants were interviewed individually. Information on their medical history and physical health conditions were gathered. Participants were also administered the Chinese version of the Beck Depression Inventory [39, 40] to assess their mood status. After the interview, EEG data were collected in a sound and light attenuated room. EEG recording started with a 5 min eyes-closed resting condition, which was followed by a 5-min *Dan Tian* Breathing (a traditional *Shaolin* mind-body exercise) condition. At baseline, participants in both the DMBI and CBT groups had no experience of *Dan Tian* Breathing. During the intervention, the DMBI but not the CBT group was trained and practiced *Dan Tian* Breathing throughout the 1-month treatment period. EEG data were recorded with 19 electrodes positioned across the scalp according to the International 10–20 System [41]. All electrode impedances were kept at 10 kΩ or below, and were referenced to linked ears. The EEG signal was digitally filtered at 0.5 and 100 Hz and sampled at 256 samples per second, with a high-frequency limit band pass of 30 Hz.

### 2.3. Intervention

The structure and format of DMBI and CBT groups were designed parallel to each other, that is, both were run as four weekly sessions of 90 mins each, had the same group size, session time and duration, with didactic teaching and learning elements, in-session sharing and discussion, and weekly home assignments.

#### 2.3.1. DMBI

The DMBI consists of four components (i) *Chan* practice, (ii) mind-body exercises, (iii) dietary monitoring and (iv) opening orifices (Figure 1). In each session of the DMBI, the participants were encouraged to change their depressogenic thoughts based upon *Chan* principles—that is, understanding that the roots of all problems were greed, anger and obsessive thinking, faulty realization of the self and nature. Participants were also encouraged to adopt a vegetarian diet and to refrain from hot and spicy food. In addition, they also learned some basic traditional *Shaolin* mind-body exercises that included training on *Dan Tian* breathing. *Dan Tian* breathing is different from common abdomen breathing. The former requires individuals to tighten their anal muscles, as well as those muscles at the abdomen and the lips when breathing out, and then to totally relax when breathing in. According to our clinical observation, this type of breathing not only helped to reduce stress but also improved the peristalsis of the guts. Also, participants were taught mind-body exercises to relax their shoulders and to massage the two sides of the nose bridge to facilitate normal breathing through the nostrils. The positive effects of DMBI on mood, neuro-electrophysiological state and bowel function are summarized in Figure 1.

#### 2.3.2. CBT

Group CBT used in the present study was based on the results of empirically validated treatments for depression [36, 42–48] which highlighted that automatic thoughts and dysfunctional attitudes were strongly related to depressive symptoms. Therefore, change in negative cognition will lead to change in depressive symptom and the program emphasized identifying, understanding and changing the negative thought and dysfunctional beliefs. During the four CBT sessions, participants learned to be aware of the symptoms of depression, the relationships among mood, cognitions and behaviors, and to identify the common cognitive distortions associated with depression. They also learned methods and procedures of cognitive restructuring, ways to improve their mood through behavioral activation and activity scheduling, and muscle relaxation techniques to reduce stress.

### 2.4. EEG Data Analyses

The computation of alpha asymmetry at the prefrontal region was well-documented and was based on the formula proposed by Davidson [37, 38], who has conducted a series of research on the use of this asymmetry index to reflect the mood state of human beings, and has repeatedly found positive association between left frontal alpha asymmetry and positive mood. Data of absolute power at the alpha frequency band (8–13 Hz) were first selected and then normalized. To normalize the distribution, alpha power values were natural logarithm (ln)-transformed [49]. EEG asymmetry was evaluated for the mid-frontal (F3, F4) pair of electrodes. EEG asymmetry scores were computed as the difference between the natural logarithm of alpha power at the right recording site (i.e., F4) and that at the left recording site (i.e., F3) [the formula is ln(F4) – ln(F3)]. Brain activity was an inverse measure of alpha power activity, meaning the higher the brain activity, the lower the alpha power, and vice versa. Consequently, positive EEG asymmetry values resulting from higher right-alpha power indicated greater left relative to right brain activity that signified positive affect, while negative values indicated the opposite brain activity pattern that signified negative affect [37].

## 3. Results

### 3.1. Behavioral Measure of Mood Changes after Treatment

#### 3.1.1. Overall Mood-Enhancing Effect

A repeated measures ANOVA was performed on BDI-II score before and after treatment (Time) between the two intervention groups. A significant main effect of Time was found [*F*(1,38) = 39.01, *P* < .001]. The two groups were comparable on the level of depressive mood at baseline as measured by their BDI-II score [*t*(19) = −0.32, *P* = .75]. Posthoc *t*-tests indicated that both the DMBI group [baseline = 14, SD = 10.42; post-test = 6.30, SD = 6.67; *t*(19) = 3.82, *P* = .001; effect size (Cohen's *d*) = 0.85], and the CBT group [baseline = 15.05, SD = 10.24; post-test = 8.90, SD = 7.91; *t*(19) = 6.66, *P* < .0005; effect size (Cohen's *d*) = 1.49] showed a significant reduction in depressive mood after treatment.

#### 3.1.2. Mood-Enhancing Effect for Moderate to Severe Depressive Mood

**Figure 2:**
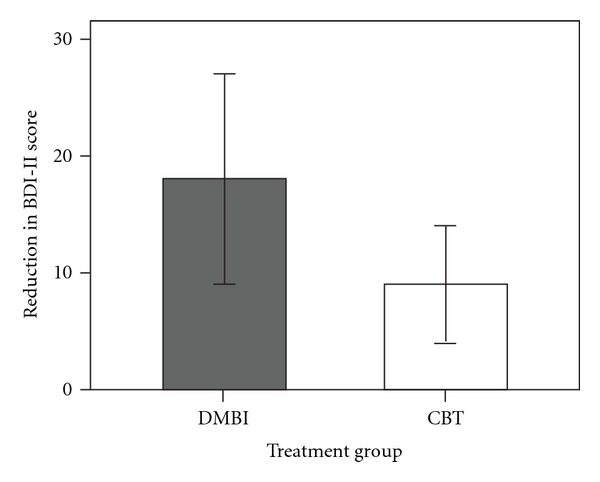
Treatment-related reduction of depressive mood within subgroups of participants with moderate or severe depressive mood at baseline. The extent of reduction for the DMBI group was significantly greater (*P<.05*) than that of the CBT group.

Given the large variation in the levels of depressive mood among the participants, subgroup analyses were conducted to examine the effect of DMBI on participants with minimal to mild degree of depressive mood, and with moderate to severe degree as shown on their baseline measurement. Results from repeated measures ANOVA showed a significant Group by Time interaction effect, *F*(1,10) = 5.04, *P* < .05. When comparing the mean difference in BDI-II score within the subgroup of moderate to severe depressive mood at baseline, the DMBI group demonstrated significantly greater treatment-related reduction of depressive mood (*n* = 6, mean reduction = 18.00, SD = 8.60) than the CBT group (*n* = 6, mean reduction = 9.00, SD = 4.73) [*t*(10) = 2.25, *P* < .05; effect size (Cohen's *d*) = 1.30] (Figure 2). No significant difference was found between the two groups on age [*t*(10) = −0.43, *P* = .68], education [*t*(10) = 0.50, *P* = .63], and level of depressive mood at baseline [*t*(10) = 0, *P* = 1]. These results suggested that DMBI, which lasted for only 1 month, seemed to be more effective than CBT for reducing the depressive mood of individuals showing more severe symptoms.

### 3.2. Electrophysiological Measure Related to Mood Changes after Treatment

#### 3.2.1. Elevated Resting Prefrontal Alpha Asymmetry

**Figure 3:**
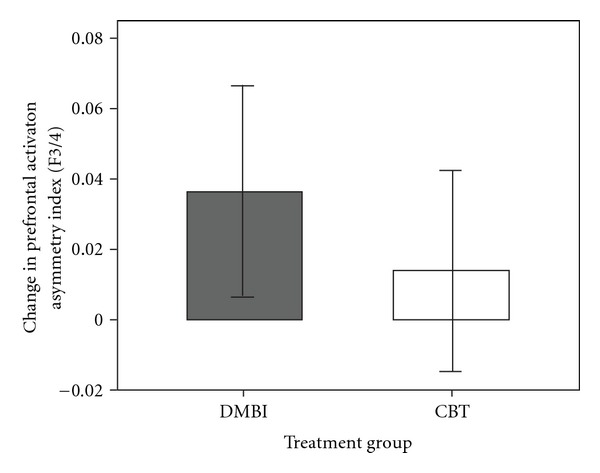
Treatment-related change in resting prefrontal activation asymmetry at the mid-frontal (F3/4) electrode site for the DMBI and the CBT group. While the DMBI group showed a significant increase (*P<.05*) in prefrontal asymmetry, the CBT group did not.

The baseline and post-intervention indices of prefrontal asymmetry at the mid-frontal site (F3/4) for each group was computed and compared. The DMBI group demonstrated a significant increase of prefrontal alpha asymmetry during eyes-closed resting condition after intervention [*t*(19) = –2.53, *P<.05*], while individuals who received CBT did not show significant change [*t*(19) = –1.01, *P=.33*].  Figure 3 presents the mean difference in the asymmetry index of each group. Thus, the results suggested that practicing the DMBI may change the electrophysiological state of the brain, which in turn is associated with increase in positive mood.

#### 3.2.2. Elevated Prefrontal Asymmetry at Dan Tian Breathing

It was hypothesized that the treatment-related change in the resting electrophysiological condition of the brain is attributable to *Dan Tian* breathing that was trained and had been practiced for 1 month in the DMBI group. Thus, the prefrontal activation asymmetry index was also recorded during each participant's performance of the breathing both at baseline and post-intervention. It should be noted that at baseline, none of the participants had practiced *Dan Tian* breathing and by the post-intervention assessment, the DMBI group had practiced this breathing for 1 month. The results indicated a significant increase [*t*(19) = −2.23, *P<.05*] in prefrontal asymmetry index among participants of the DMBI group at the post-intervention assessment (mean = 0.064, SD = 0.052) compared with that at baseline (mean = 0.032, SD = 0.058), but not for the CBT group [post-intervention mean = 0.057, SD = 0.042; baseline mean = 0.042, SD = 0.044; *t*(19) = −1.19, *P=.25*] who had not practiced *Dan Tian* breathing. These results supported our hypothesis that *Dan Tian* breathing practice may change the activity of the brain associated with enhanced positive mood.

### 3.3. Changes in Bowel Function after Treatment

#### 3.3.1. Self-Reported Improvement in Bowel Function

As it has been shown in our prior clinical observation on patients that the practice of *Dan Tian* breathing helped to improve digestive functions, we attempted to examine this phenomenon qualitatively and quantitatively in the present study. First, in the post-intervention assessment, each participant was asked to rate their change of bowel condition on a 6-point scale (3, 2, 1, 0, −1, −2, −3) with 0 indicating no change, 3 the greatest improvement and −3 the greatest deterioration. While participants of the DMBI group showed a mean rating of 1.95 points in the direction of improvement, those in the CBT group showed a mean rating of 0.25 indicating no apparent improvement. This difference between the two groups was significant [*t*(38) = 2.57, *P<.05*], suggesting that participants in the DMBI but not the CBT group showed subjective improvement in bowel condition. In addition, while 55% of the DMBI group of participants reported improvement in bowel function, only 15% of the CBT group reported such improvement.

#### 3.3.2. Quantitative Improvement in Bowel Function

In order to quantify the improvement, more objective information was obtained in the interviews at baseline and post-intervention. Three important indicators of healthy bowel function were used, including the frequency of bowel movement per day, the time needed to empty the bowel, and number of days per week that the participants felt their bowel was not completely emptied after bowel movement. Participants were asked to recall their average frequency or duration, in respect of the three indicators, in the previous month. For the DMBI group, significant improvement was found on both the amount of time for emptying the bowel [*t*(19) = 2.54, *P<.05*], and feeling of incomplete emptying of the bowel after a bowel movement [*t*(19) = 2.10, *P<.05*]. For the CBT group, no significant change on any aspect of bowel was reported (Table 2). These results provided further support to our prior clinical observation that the DMBI helped improve constipation, and that the effect was likely to be attributable to the practice of *Dan Tian* breathing.

## 4. Discussion

While the majority of psychological interventions require months of treatment before any effect can be observed, the major findings of this study suggested that DMBI may have a significant mood-enhancing effect on community-dwelling adults with depressive mood even with just 1 month of training. Specifically, individuals with moderate to severe depressive mood who have participated in DMBI demonstrated significant reduction in depressive mood compared with those who participated in CBT. The lack of significant improvement in the latter group may probably be attributable to the inadequate duration of treatment to derive optimal treatment effects, which usually takes more than 1 month [36]. With short-term intervention, the DMBI seemed to be more effective than CBT in improving depressive mood. While DMBI has demonstrated its relative strength as a brief intervention for reducing stress and improving mood, the therapeutic effect of a longer term DMBI intervention remains to be examined.

An alternative explanation for the apparently greater treatment-related improvement for participants with more severe depressive mood in the DMBI group may be resulting from the statistical artifact of regression to the mean through repeated measures. However, randomization, together with the fact that the magnitude of treatment-related improvement in mood for DMBI participants was double that of the CBT participants, provided evidence against the attribution of such observed improvement to a statistical phenomenon [50].

On the self-reported rating of mood using BDI-II, both the DMBI and the CBT groups showed a significant treatment-related reduction of depressive mood. However, only participants of the DMBI but not the CBT group showed a significant leftward shift of the frontal asymmetry index after treatment, which might signify a significant increase in positive affect/mood. These discrepant results between the self-reported measure of mood (BDI-II) and the objective, neurophysiological measure of affect/mood suggested that the two outcome measures might be measuring different aspects of an affective phenomenon. That is, the neurophysiological change may precede the subjective feeling as reported by the participants. Alternatively, the self-report may be biased [51, 52]. Thus, findings from the present study supports the notion that the inclusion of neurophysiological measures for evaluating changes in affect may be particularly important for studies that examine the effect of intervention involving meditative practices, an area in which the associated neurophysiological changes have been well-documented [53].

Participants of the DMBI reported significant treatment-related improvement in their bowel condition, while no such improvement was reported among participants of CBT. Consistent with the self-reports, the quantified interview data revealed that out of the three parameters of bowel condition, significant improvements were found on two (i.e., amount of time for emptying the bowel, and the feeling of incomplete emptying of the bowel after defecation) in the DMBI group, while no improvement was found in the CBT group. These results provided initial empirical evidence suggesting that the DMBI has positive effects on both the mind (i.e., depressive mood) and the body (i.e. bowel condition). It should be noted that the improvement of bowel function has not yet been reported as a treatment outcome for most psychological therapies; thus the mind-body intervention approach seems to have an additional therapeutic effect besides psychological intervention.

Qualitative analyses of the feedback from participants of the DMBI group revealed that this form of treatment was well-received. On the one-item forced choice question that tapped the acceptability of the treatment, 96% of the DMBI participants rated treatment as helpful for enhancing their health, while 62% of the CBT participants found the treatment helpful. In sum, participants of the DMBI group regarded the treatment as helpful in relieving their anxiety, and improving their mood. Besides, these participants also reported benefits on their cognitive functioning, such as helping them to feel more alert, refreshed, or attentive/concentrated. Benefits on the spiritual aspects, in terms of increased self-reflection and awareness, were also reported. One of the participants even reported that he quitted smoking successfully during his participation in the DMBI.

Despite this initial evidence that suggested that DMBI is an effective treatment for relieving depressive mood, some limitations of the current study are worth noting. The majority of participants recruited in the study were in a narrow age range (between 40 and 55 years), and were relatively well-educated compared with peers of their age. In addition, while findings of the current study indicated that DMBI was effective in alleviating depressive mood of a community-dwelling adult sample, the applicability and effectiveness of its mood-enhancement effect on patients with clinical depression need further investigation. It should also be noted that volunteers came from attendees to a public lecture on *Shaolin Chan*, which suggested that they may be pre-inclined towards the intervention. Interpretation of the results should therefore be made in light of this. In addition, the fact that DMBI might be more culturally acceptable and familiar to Chinese participants than it is to participants in the West should be borne in mind when planning treatment for non-Chinese patients. It should also be noted that the tool for measuring bowel function was not a standardized tool. Future studies using standardized tools to assess bowel function would be very helpful.

In sum, given the encouraging results from the present study on the health benefits of the DMBI, future studies are needed to replicate and extend the findings with the use of a larger sample from a wider age range and educational level, and to patient groups with clinical depression. Given that the DMBI is easy to learn, convenient to practice, and can be delivered in large groups such as talks and workshops, its potential use as a primary prevention program (e.g., for depression) in the community deserves further investigation.

## Figures and Tables

**Table 1:** Demographic characteristics of participants.

Characteristics	DMBI group		CBT group			
	(*n=20*)		(*n=20*)	
	Mean	SD	Mean	SD	*t-*value	*P*-value
Age (years)	49.65	7.27	48.92	8.11	0.30	.76
Education (years)	13.00	2.61	12.85	2.72	0.18	.86
Depressive Mood	14.00	10.42	15.05	14.24	−0.32	.75
(BDI-II)						
Gender—female (%)	66.67		66.67			

DMBI: *Dejian* Mind-Body Intervention; CBT: Cognitive-Behavioral Therapy; BDI-II: Beck Depression Inventory-version II.

**Table 2:** Comparisons on the pre- and post-treatment scores on the three parameters of bowel condition of the two treatment groups.

Three items on bowel condition	Pre-treatment	Post-treatment	*t-*value	*P-*value
Treatment group	Mean (SD)	Mean (SD)		
Frequency of bowel movement (per day)				
DMBI group	1.17 (.37)	1.31 (0.58)	*−1.32*	.20
CBT group	1.15 (.61)	1.16 (.67)	*−0.08*	.94
Amount of time for emptying bowel (min)				
DMBI group	5.90 (3.22)	4.73 (1.82)	2.54*	.02
CBT group	6.33 (3.56)	5.45 (2.55)	1.14	.27
Feeling of incompletely emptied bowel				
after bowel movement (no. of days/week)				
DMBI group	1.68 (1.69)	0.95 (1.09)	2.10*	.049
CBT group	1.13 (1.74)	1.23 (2.13)	*−0.35*	.73

*P<.05; DMBI: *Dejian* Mind-Body Intervention; CBT: Cognitive-Behavioral Therapy.
